# Antibiotic trends of *Klebsiella pneumoniae* and *Acinetobacter baumannii* resistance indicators in an intensive care unit of Southern Italy, 2008–2013

**DOI:** 10.1186/s13756-015-0087-y

**Published:** 2015-11-03

**Authors:** Antonella Agodi, Martina Barchitta, Annalisa Quattrocchi, Andrea Maugeri, Eugenia Aldisio, Anna Elisa Marchese, Anna Rita Mattaliano, Athanassios Tsakris

**Affiliations:** Department of Medical and Surgical Sciences and Advanced Technologies “GF Ingrassia”, University of Catania, Via S. Sofia 87, 95123 Catania, Italy; Azienda Ospedaliero-Universitaria ‘Policlinico-Vittorio Emanuele’, Via S. Sofia, 78, 95123 Catania, Italy; Department of Microbiology, Medical School, University of Athens, 75 Mikras Asias Street, 11527 Athens, Greece; Department “GF Ingrassia”, University of Catania, via S. Sofia, 87, 95123 Catania, Italy

**Keywords:** Antimicrobial resistance, Resistance rates, Antimicrobial usage density

## Abstract

**Background:**

The overuse of antimicrobials is one of the main factors responsible for the development and spread of antimicrobial resistance, together with other causes, such as intra- and inter-hospital spread of resistant microorganisms and infection control policies and practices. The objective of the present study is to report the trends of *Klebsiella pneumoniae* and *Acinetobacter baumannii* antimicrobial resistance indicators in an Italian intensive care unit (ICU) during a six-year period, from 2008 to 2013.

**Methods:**

Susceptibility data and annual antibiotic consumptions in the ICU were retrospectively obtained from the clinical laboratory and the pharmacy. Trends over time of resistance rates (RRs) and of incidence densities of resistant isolates were determined by linear regression.

**Results:**

Isolation density of *A. baumannii* increased significantly from 2008 (20.4 *per* 1,000 patient-days) to 2013 (58.1 *per* 1,000 patient-days) and of *K. pneumoniae* from 2010 (22.3 *per* 1,000 patient-days) to 2013 (55.9 *per* 1,000 patient-days). RRs of third-generation cephalosporins (3GCs)-resistant *K. pneumoniae* (from 2010: 41.9 %, to 2012: 87.0 %), of carbapenem-resistant *K. pneumoniae* (from 2008: 0 %, to 2013: 59.2 %), and of carbapenem-resistant *A. baumannii* (from 2008: 87.5 %, to 2013: 96.6 %) showed significant increasing trends. Carbapenems was the main antibiotic class consumed (24.9 % of the total antimicrobial usage density), followed by 3GCs (21.0 %), fluoroquinolones (20.6 %), aminoglycosides (17.3 %), penicillins (15.1 %) and glycopeptides (1.1 %). Carbapenems consumption decreased from 2008 to 2012 and then increased in 2013. Glycopeptides consumption decreased from 2008 to 2011 and then increased in 2013. Aminoglycosides consumption decreased from 2008 to 2010 and increased from 2012 to 2013. Finally, 3GC, penicillins and fluoroquinolones consumptions decreased from 2012 to 2013.

**Conclusions:**

RRs of carbapenem-resistant *A. baumannii* and of carbapenem- and 3GC-resistant *K. pneumoniae* were higher than those for Europe. Our findings highlight the necessity to implement an integrated system for monitoring not only consumption of antibiotics and resistance profiles but also the clonality of alert microorganisms in the ICU for effective infection control.

## Background

Antimicrobial resistance (AMR) is a severe threat to public health in Europe and worldwide, leading to growing costs, treatment failure, and mortality [1, 2]. AMR results in reduced efficacy of drugs, and limits the available treatment options. The magnitude of the problem worldwide, the impact of AMR on health and on costs for the healthcare sector, together with the societal impact, are still unknown [2]. Thus, surveillance of AMR is considered an essential component of an effective response to this problem, and results produced constitute a fundamental source of information on the burden and trends of resistance [1]. Unfortunately, high resistance rates (RRs) to antimicrobial agents have been observed among bacterial pathogens that cause healthcare-associated and community-acquired infections worldwide and significant gaps in surveillance, lack of standards for methodology, data sharing and coordination have been highlighted [2]. Especially Gram-negative bacteria, including multidrug resistant *Acinetobacter baumannii* and *Enterobacteriaceae* producing Extended-spectrum beta-lactamases (ESBL) and carbapenemases, have been associated to severe healthcare-associated infections and their occurrence has increased in the last decades [1, 3]. In Europe, the most recent report of the European Antimicrobial Resistance Surveillance Network (EARS-Net) describes a general increase of AMR in the Gram-negative pathogens including *Klebsiella pneumoniae* and *A. baumanni* [1]. In Italy, the results of the Italian Nosocomial Infections Surveillance in Intensive Care Units (SPIN-UTI) network revealed that *K. pneumoniae* and *A. baumanni* are among the most commonly isolated microorganisms in intensive care unit (ICU)-acquired infections [4–6].

The overuse of antimicrobials is one of the main factors responsible for the development and spread of AMR. Therefore, European countries increasingly implement actions to control AMR in the community and the hospital setting through rational use of antimicrobials. Information on antimicrobial consumption in Europe is a prerequisite for antibiotic stewardship and can be an important source for healthcare professionals and policy makers in order to monitoring progress towards a prudent use of antibiotics [7]. However, other factors, such as intra- and inter-hospital spread of resistant microorganisms, community contribution to resistance, and infection control policies and practices, may also play a role in determining the burden of resistance in a hospital and should be considered [8]. We have previous reported intra- and inter-hospital spread of *K. pneumoniae* clones in two Sicilian ICUs [9] and the emergence of a carbapenem-resistant *K. pneumoniae* clone in one of those ICUs [10]. Furthermore, recently, we have described the dissemination, in the two ICUs of clonally related isolates of carbapenem-resistant *A. baumannii* with simultaneous resistance to colistin, hypothesizing that prior carbapenem and colistin consumptions may have acted as triggering factors [11].

All these findings support the need of strict adherence to control measures to prevent the dissemination of multidrug-resistant microorganism, together with the monitoring of antibiotic consumption at local level to promote the judicious use of antibiotics. The objective of the present study is to report the trends of *K. pneumoniae* and *A. baumannii* resistance indicators in an Italian ICU during a six-year period, from 2008 to 2013.

## Methods

### Setting

A retrospective study was conducted, during the period 2008–2013, at an 8-bed interdisciplinary ICU of the Azienda Ospedaliero – Universitaria “Policlinico - Vittorio Emanuele” in Catania, Italy. The total number of patient-days for each year was collected at the unit level.

### Microorganisms and antibiotic susceptibility tests

Data on microorganisms isolated from ICU patients were retrospectively obtained from the clinical laboratory of the hospital. Identification and susceptibility testing, including ESBL test, were performed using the Phoenix System (Becton Dickinson, Sparks, MD, USA). A laboratory-based surveillance, without patient-based data collection, was performed and the susceptibility data were collected for *K. pneumoniae* and *A. baumannii* regardless of whether they were associated with healthcare-associated or community-acquired infection or colonization, or whether they were from clinical or surveillance cultures. Particularly, data on carbapenem and 3GC -resistant *K. pneumoniae* and on carbapenem-resistant *A. baumannii* were included in the present study. Isolates were classified as susceptible, intermediate or resistant according to Clinical and Laboratory Standards Institute guidelines [12] and, from 2012, to European Committee on Antimicrobial Susceptibility Testing guidelines [13]. Copy strains, defined as an isolate of the same species showing the same susceptibility pattern throughout a period of 1 month in the same patient, no matter what the site of isolation, were excluded [14]. For each species, the isolation density was calculated as the number of isolates *per* 1,000 patient-days. Antibiotic RRs were calculated as the number of resistant or intermediate isolates divided by the total number of isolates of the same species tested against the corresponding antibiotic multiplied by 100. Furthermore, the incidence density of resistant isolates was calculated as the number of resistant or intermediate isolates *per* 1,000 patient-days.

### Antibiotic consumption

Data on annual antibiotic consumption in the ICU, from 2008 to 2013, were obtained from the pharmacy of the hospital. Consumption - that is the antimicrobial usage density (AD) – was expressed as defined daily dose (DDD) and was normalized *per* 1,000 patient-days. The DDD are the standard adult daily dose of an antimicrobial agent for a 1-day treatment defined by the World Health Organization [15]. Particularly, data on consumption of third-generation cephalosporins (3GCs: ceftriaxone, cefotaxime and ceftazidime), penicillins (piperacillin, piperacillin/tazobactam, ampicillin, amoxicillin), glycopeptides (vancomicin), carbapenems (imipenem, meropenem, and ertapenem), aminoglycosides (amikacin, gentamicin, tobramicin and netalmicin), and fluoroquinolones (levofloxacin and ciprofloxacin) were collected. Notably, gentamicin and ertapenem were introduced into the ICU in 2009 and 2011, respectively.

### Statistical analyses

Statistical analyses were performed using the SPSS 22.0 statistical package (SPSS Inc., Chicago, IL, USA). Trends over time of RRs and of incidence densities of resistant isolates were determined by linear regression with the yearly data. Furthermore, rate ratios were computed to compare ADs between different years. Pearson’s correlation coefficient (cc) was used to determine the relationship between antibiotic consumption and antibiotic RRs and incidence densities of resistant isolates. A p value <0.05 was considered statistically significant.

## Results

### Microorganisms and antibiotic resistance

During the six-year period, among the 5,529 non-repetitive microbial isolates recovered from clinical or surveillance samples, Gram-negatives were the most frequently isolated microorganisms (48.6 %), followed by fungi (29.6 %) and Gram-positives (21.8 %). Particularly, a total of 475 *K. pneumoniae* (8.6 %) and of 463 *A. baumannii* (8.4 %) were isolated. Isolation density of *A. baumannii* increased significantly from 2008 (20.4 *per* 1,000 patient-days) to 2013 (58.1 *per* 1,000 patient-days) and of *K. pneumoniae* from 2010 (22.3 *per* 1,000 patient-days) to 2013 (55.9 *per* 1,000 patient-days) (Table 1).

**Table 1:** Isolation density, resistance rates and incidence density of resistant isolates during the period 2008 – 2013

	Year						
	2008	2009	2010	2011	2012	2013	*p*-value*
**Isolation density**							
**(** ***per*** **1000 patient-days)**							
*Klebsiella pneumoniae*	38.0	48.5	22.3	31.9	41.6	55.9	0.497 (**0.005**, from 2010 to 2013)
*Acinetobacter baumannii*	20.4	19.3	29.8	49.0	50.3	58.1	**0.002**
**Resistance rates, %**							
**(number of tested isolates)**							
3GC-resistant *K. pneumoniae*	88.7 (53)	69.4 (62)	41.9 (43)	67.2 (64)	87.0 (69)	81.6 (98)	0.806 (**0.045** from 2010 to 2012)
ESBL-producing *K. pneumoniae* among 3GC-resistant *K. pneumoniae*	100	100	100	44.2	98.3	93.8	0.674
	(39)	(42)	(18)	(43)	(59)	(80)	
Carbapenem-resistant *K. pneumoniae*	0 (53)	9.7 (62)	2.3 (43)	37.5 (64)	63.8 (69)	59.2 (98)	**0.010**
Carbapenem resistant *A. baumannii*	87.5 (14)	95.0 (19)	96.2 (51)	98.9 (92)	93.2 (68)	96.6 (85)	**0.021**
**Incidence density of resistant isolates (** ***per*** **1,000 patient-days)**							
3GC-resistant *K. pneumoniae*	25.9	25.1	8.9	19.3	32.8	35.5	0.367 (**0.027** from 2010 to 13)
ESBL-producing *K. pneumoniae* among 3GC-resistant *K. pneumoniae*	21.5	24.6	8.9	8.5	31.7	33.2	0.436
Carbapenem-resistant *K. pneumoniae*	0	3.5	0.5	10.8	24.1	25.7	**0.009**
Carbapenem-resistant *A. baumannii*	7.7	11.1	25.3	41.3	37.2	37.7	**0.014**

*Linear regression

Significant values are indicated in bold font

*Abbreviation*: *3GC* third-generation cephalosporin, *ESBL* Extended-spectrum beta-lactamase

Table 1 shows the RRs during the study period. RRs of 3GC-resistant *K. pneumoniae* (from 2010: 41.9 %, to 2012: 87.0 %), of carbapenem-resistant *K. pneumoniae* (from 2008: 0 %, to 2013: 59.2 %), and of carbapenem-resistant *A. baumannii* (from 2008: 87.5 %, to 2013: 96.6 %) showed significant increasing trends. Frequencies and incidence densities (*per* 1,000 patient-days) of ESBL-producing *K. pneumoniae* among 3 GC-resistant *K. pneumoniae* were shown in Table 1.

Considering incidence density of resistant isolates (*per* 1,000 patient-days), significant increasing trends were reported for 3GC-resistant *K. pneumoniae* (from 2010 to 2013), carbapenem-resistant *K. pneumoniae* (from 2008 to 2013), and carbapenem-resistant *A. baumannii* (from 2008 to 2013) (Table 1).

### Antibiotic consumption

**Fig. 1:**
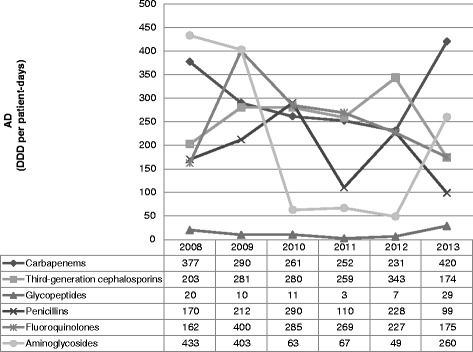
Annual consumption of antibiotic classes from 2008 to 2013

Antibiotic consumption over the 6-year period was 7,362 DDD *per* 1,000 patient-days (AD). Overall carbapenems (24.9 % of the total AD) was the main antibiotic class consumed, followed by 3GCs (21.0 %), fluoroquinolones (20.6 %), aminoglycosides (17.3 %), penicillins (15.1 %) and glycopeptides (1.1 %). Figure 1 shows the heterogeneity of the antibiotic consumption (in AD), by antibiotic classes, and the time-trend distribution in the ICU.

In general, the consumption of each antibiotic class varied with years. Carbapenems consumption decreased from 2008 to 2012 (rate ratio 0.61; 95 % CI 0.54–0.69) and then increased, by about 2-fold, in 2013 (rate ratio 1.81; 95 % CI 1.61–2.02). Glycopeptides consumption decreased from 2008 to 2011 (rate ratio 0.15; 95 % CI 0.06–0.35) and then intensely increased in 2013 (rate ratio 10.85; 95 % CI 4.73–30.63). Aminoglycosides consumption decreased from 2008 to 2010 (rate ratio 0.15; 95 % CI 0.12–0.18) whereas it increased from 2012 to 2013 (rate ratio 5.34; 95 % CI 4.27–6.76). Finally, 3GCs (rate ratio 0.51; 95 % CI 0.45–0.58), penicillins (rate ratio 0.44; 95 % CI 0.37–0.52) and fluoroquinolones (rate ratio 0.77; 95 % CI 0.67–0.89) consumptions decreased from 2012 to 2013.

**Fig. 2:**
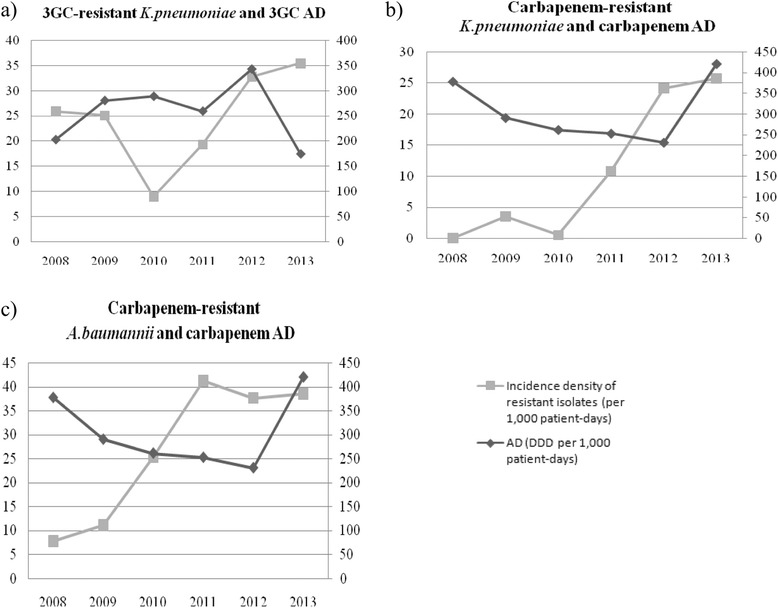
Incidence density of resistant isolates and antibiotic consumption during 2008–2013. **a**. 3GC - resistant *K. pneumoniae* and 3GC AD. **b**. Carbapenem – resistant *K. pneumoniae* and carbapenem AD. **c**. Carbapenem – resistant *A. baumannii* and carbapenem AD. Abbreviation: 3GC, third-generation cephalosporin, AD: antimicrobial usage density; DDD: defined daily dose

Figure 2 reports trends of incidence density of resistant isolates (*per* 1,000 patient-days) and of the corresponding antibiotic consumption (in AD) during the study period. No correlations between the usage of antimicrobial agents and resistance data were found.

## Discussion

Italy is one of the European countries with increasing spread of antimicrobial-resistant microorganisms, often multidrug-resistant [1] and with high antibiotic consumption in the hospital setting [1, 7, 16]. Several factors, such as antimicrobial consumption, clonal spread of resistant microorganisms, resistance mechanisms that might differ by species, the human and environmental reservoir, and infection control strategies, including screening policies, may play a role in the prevalence of antimicrobial-resistant pathogens in the hospital setting [8, 14]. This issue is of interest especially in Italian ICUs where the highest prevalence of patients on antibiotic treatment is observed [17] and outbreaks due to multidrug-resistant *K. pneumoniae* and *A. baumannii* are frequently reported [9–11, 18]. Comparison of our data with those of the EARS-Net should be undertaken with caution, since European results are based on invasive isolates from blood or cerebrospinal fluid, thus not representative of isolates from other sites [1]. Our study confirms that carbapenem resistance is endemic for *A. baumannii* isolates, with dramatically higher RRs (about 95 %) than those in the other European countries [1]. Furthermore, incidence density of this multidrug-resistant pathogen increased significantly from 2008 to 2013 in our ICU, and consumption of carbapenems, after a significant decrease, increased in the last year of the study. Recently, it has been reported that the increasing use of carbapenems was associated with the increasing incidence of healthcare-associated infection due to imipenem-resistant *A. baumannii,* suggesting that caution in antibiotic use would play an important role in managing high RRs [19]. The results of our study, although not confirmatory, may also suggest that increasing carbapenems consumption contributed to increasing rate of drug-resistant organisms. However, in order to further explain high RRs of pathogens associated with infections in the ICUs, it has been suggested [11, 20, 21] that high RRs are correlated with high rates of antibiotic use, but increased and/or high RRs may also be due to transmission between patients. In fact, in the same ICU, we have recently described the dissemination of clonally related multidrug-resistant *A. baumannii* isolates [11].

In Europe, the percentage of carbapenem-resistant *K. pneumoniae* is already high and increasing in some countries. The highest RRs were reported by Greece followed by Italy (28.8 %) with statistically significant increasing trends [1]. Since carbapenem- resistant *K. pneumoniae* isolates are frequently found to be carbapenemase-producing, these results highlight and confirm that surveillance, active screening of patients at high-risk, notification of health authorities, implementation of infection control measures and the prudent use of antimicrobials are key elements to contain the spread of carbapenem-resistant *K. pneumoniae* isolates [1, 22, 23]. In Italy since 2013 the Ministry of Health established a national surveillance of carbapenemase-producing *Enterobacteriaceae* (particularly, *K. pneumoniae* and *Escherichia coli*) [24]. In our ICU the percentage of carbapenem-resistant *K. pneumoniae* was higher than that one documented in Europe with a significant increasing trend, although consumption of carbapenems decreased until 2012 and increased in 2013. As described for *A. baumanni*, dissemination of clonally related carbapenem-resistant *K. pneumoniae* isolates has been also previously documented in our unit, thus contributing to the increasing trend of carbapenems resistance [9, 10]. Notably, results on high carbapenem RRs of *A. baumannii* and *K. pneumoniae* are worrisome because carbapenems are last-line antibiotics. In addition, as previously reported, when carbapenems resistance is due to the presence of carbapenemases, accumulation of other resistance determinants, makes these isolates extensively drug- or pandrug-resistant, leaving few or no effective treatment options [22, 23, 25, 26].

In European countries, a large proportion of 3GC-resistant *K. pneumoniae* isolates, ranging between 85 % and 100 %, was ascertained as ESBL-positive [1]. In our study, frequencies of ESBL-producing *K. pneumoniae* among 3GC-resistant isolates were very high, reaching 100 % in 2008, 2009 and 2010. Furthermore, the incidence density of ESBL producers increased, although not significantly, from 2010 to 2013. The high percentage of ESBL-producing *K. pneumoniae* complicates the treatment of serious infections caused by these bacteria leading to a significant increase in carbapenems use, considered the first line class for ESBL producer, with a consequent impact on the emergence of resistance to these antibiotics [1].

In our ICU the percentage of 3GC-resistant *K. pneumoniae* isolates was higher than that one reported for Italy (47.7 %) [1]. Furthermore, the incidence density of resistant isolates increased significantly from 2010 to 2012 together with the 3GC consumption. In 2013, the 3GC consumption decreased but the incidence density of resistant isolates increased with no evidence of clonal dissemination of 3GC-resistant *K. pneumoniae* in the ICU. Interestingly, in the German ICUs the dramatic increase of 3GC-resistant *K. pneumoniae* has been associated with an increased trend of antibiotic usage [14].

Finally, in the present study no correlations between resistance data and the corresponding antibiotic consumption were observed, thus the multifaceted nature of the spread and emergence of resistance can, at least partly, explain this result. In fact, although it is clear that antibiotics may act as promoters of resistance inducing key processes for the emergence and spread of resistance, as mutagenesis, recombination and/or horizontal gene transfer [8, 14, 19, 27], this occurrence is also influenced by other factors than by antibiotic consumption alone. Antibiotic-resistant bacteria, as well as resistance genes, can spread from person to person to the environment, and then back to humans thus, infection prevention and control activities to limit the spread of resistant bacteria are crucial [28].

Furthermore, although DDD measurements are useful for comparison and benchmarking, they may not fully correlate with subsequent antibiotic resistance development due to the intrinsic biases [29].

Our study has the limitation of the retrospective study design as well as the fact that is conducted in a single institution in Italy, thus the results cannot be representative of all ICUs of Southern Italy. Moreover, the ecological study design cannot prove a causative relationship between the two factors. Thus, a prospective multicentre patient-based study design is needed to confirm these findings. Furthermore, in our study design, data on AMR are laboratory-based, thus precluding a patient-based evaluation (i.e. date of admission to the ICU, clinical data confirming infection and other information) to differentiate whether microorganisms were associated with healthcare-associated or community-acquired infection or colonization. Since the emergence of drug-resistant microorganisms is commonly seen in nosocomial settings and invasive isolates could be of much more interest than microorganisms isolated from colonization episodes, microorganisms associated with infection episodes should be analyzed separately. Besides, no data about other factors such as patient’s characteristics and the amount of antibiotics prescribed in the outpatient setting and potential confounders, such as antimicrobial stewardship interventions, were taken into account. Finally, outbreaks not investigated might have influenced resistance data.

## Conclusions

The already high percentages and increasing trends of AMR *K. pneumoniae* and *A. baumannii,* described in this study, illustrate the continuous loss of effective antimicrobial therapy against these microorganisms and emphasize the need for comprehensive strategies targeting prudent use of antibiotics, since treatment options for infections with multiresistant bacteria are limited [1]. Besides, since very few effective antibiotics for multidrug-resistant Gram-negatives pathogens are likely to be launched in a close future, there is a critical need to implement strategies against the development of acquired resistance [30]. In our study, although increasing trends of AMR organisms and of antibiotic consumption were described, we did not confirm that increasing antibiotic consumption was correlated to increasing RRs, but we suggest that the introduction of phenotypic and molecular methods for enhanced surveillance and outbreak detection may result in improved infection control programs. Our findings also recommend the implementation of an integrated system for monitoring not only consumption of antibiotics and resistance profiles but also the clonality of alert microorganisms in the ICU for effective infection control.

## Abbreviations

3GC: third-generation cephalosporins
AD: antimicrobial usage density
AMR: antimicrobial resistance
cc: correlation coefficient
DDD: defined daily dose
EARS-Net: european antimicrobial resistance surveillance network
ESBL: extended-spectrum beta-lactamases
ICU: intensive care unit
RRs: resistance rates
